# Electrochemical Sensing of Lead Ions Using Ionophore-Modified Raspberry-like Fe_3_O_4_–Au Nanostructures via Differential Pulse Voltammetry

**DOI:** 10.3390/polym17223015

**Published:** 2025-11-13

**Authors:** Giang Huong Dau, Tin Phan Nguy, Tram Thi Ngoc Do, Thanh Van Pham, Lien Thi Ngoc Truong

**Affiliations:** 1 Convergence Technology Division, Vietnam-Korea Institute of Science and Technology, Hoa Lac High-Tech Park, Km29 Thang Long Boulevard, Hanoi 100000, Vietnam; dhuonggiang@mic.gov.vn; 2 Faculty of Engineering Physics, Hanoi University of Science and Technology, Hanoi 100000, Vietnam; tin.nguyphan@hust.edu.vn (T.P.N.);; 3 Faculty of Physics, Hanoi University of Science, Vietnam National University, Hanoi 100000, Vietnam

**Keywords:** raspberry-like nanostructures, electrochemical sensor, detection of lead ions

## Abstract

This study presents the design and application of an electrochemical sensor for selective detection of lead ions (Pb^2+^) based on ionophore-modified raspberry-like Fe_3_O_4_–Au nanostructures. The material was engineered with a magnetic Fe_3_O_4_ core, coated with polyethyleneimine (PEI) to facilitate nucleation, and subsequently decorated with Au nanoparticles, providing a raspberry-like (Fe_3_O_4_@PEI@AuNPs) nanostructure with high surface area and excellent electrochemical conductivity. Surface functionalization with Lead Ionophore IV (ionophore thiol) introduced Pb^2+^-selective binding sites, whose presence and structural evolution were verified by TEM and Raman spectroscopy. The Fe_3_O_4_ core endowed strong magnetic properties, enabling facile manipulation and immobilization onto screen-printed carbon electrodes (SPCEs) via physical adsorption, while the Au nanoparticles enhanced electron transfer, supplied thiol-binding sites for stable ionophore anchoring, and increased the effective electroactive surface area. Operational conditions were systematically optimized, with acetate buffer (HAc/NaAc, pH 5.7) and chronoamperometric preconcentration (CA) at −1.0 V for 175 s identified as optimal for differential pulse voltammetry (DPV) measurements. Under these conditions, the sensor exhibited a linear response toward Pb^2+^ from 0.025 mM to 2.00 mM with superior sensitivity and reproducibility compared to conventional AuNP-modified SPCEs. Furthermore, the ionophore-modified Fe_3_O_4_–Au nanostructure-based sensor demonstrated outstanding selectivity for Pb^2+^ over competing heavy metal ions (Cd^2+^, Hg^2+^, Cr^3+^), owing to the specific coordination interaction of Lead Ionophore IV with target ions. These findings highlight the potential of raspberry-like Fe_3_O_4_@PEI@AuNP nanostructures as a robust and efficient electrochemical platform for the sensitive and selective detection of toxic heavy metal ions.

## 1. Introduction

Lead (Pb^2+^) is one of the most hazardous heavy metal ions, commonly released into the environment through industrial activities such as mining, metallurgy, and battery manufacturing. Due to its non-biodegradable nature, Pb^2+^ can persist and accumulate in water and soil over long periods, posing serious risks to ecosystems and human health, including neurological disorders, cognitive impairment in children, and kidney dysfunction in adults. The World Health Organization (WHO) has set the maximum permissible concentration of lead in drinking water at 10 μg/L, while the U.S. Environmental Protection Agency (EPA) establishes an action level of 15 μg/L for public water systems [1,2]. Therefore, monitoring Pb^2+^ concentrations in environmental and food samples has become an urgent scientific and societal necessity. In recent years, numerous studies have focused on the application of nanotechnology in the water treatment, adsorption, and detection of heavy metal ions, particularly Pb^2+^ [3,4,5].

Conventional analytical techniques such as atomic absorption spectroscopy (AAS) and inductively coupled plasma mass spectrometry (ICP–MS) remain the gold standards for heavy metal detection due to their high sensitivity and accuracy. However, these methods require expensive instrumentation, complicated sample preparation, long analysis times, and are unsuitable for on-site measurements. In contrast, electrochemical techniques such as anodic stripping voltammetry (ASV) and differential pulse voltammetry (DPV) have emerged as promising alternatives, offering high sensitivity, low cost, operational simplicity, and excellent potential for miniaturization [6,7,8]. Nevertheless, traditional electrochemical sensors still face challenges such as overlapping metal ion peaks, limited selectivity, and signal degradation after repeated use, primarily due to poor electrode surface stability.

To overcome these limitations, the integration of nanomaterials into electrochemical sensors has proven to be an effective approach to significantly enhance analytical performance. For instance, gold nanoparticles (AuNPs) provide high electrical conductivity, efficient electron transfer, and an enlarged active surface area, facilitating the immobilization of functional molecules [9,10,11]. The Fe_3_O_4_ magnetic core enables facile manipulation and concentration of the sensing material on the electrode surface under a weak external magnetic field, thereby increasing Pb^2+^ accumulation within the sensing region and enhancing the DPV signal intensity. Conductive polymers such as polyaniline (PANI) and poly(3,4-ethylenedioxythiophene) (PEDOT) not only serve as binding matrices for nanoparticles, but also improve the mechanical, chemical, and electrochemical stability of electrodes [12,13]. Recent studies, such as that by Zepeda-Navarro et al. (2024), have comprehensively reviewed the potential of multi-ion imprinted polymers (MIIPs) for heavy metal detection, emphasizing the importance of optimizing porous structures and ion selectivity [14]. Similarly, Zdorovets et al. (2019) demonstrated the effectiveness of UV-modified poly (ethylene terephthalate) (PET) membranes grafted with amine- and carboxyl-containing copolymers for simultaneous detection of Cu^2+^, Pb^2+^, and Cd^2+^ at microgram-per-liter levels [15]. Abdulla et al. (2019) also developed a double-thiol-linked PProDOT@Si-based electrochemical sensor capable of simultaneously detecting Cd^2+^, Pb^2+^, and Hg^2+^ with nanomolar detection limits [16]. Furthermore, Lu et al. (2021) reported that coating conventional graphite electrodes with PEDOT significantly improved active surface area, electron transfer, and signal stability [17].

Another crucial factor in designing ion-selective sensors is the ionophore—molecules capable of forming specific complexes with target ions through coordination interactions. For Pb^2+^, Lead Ionophore IV (tert-Butylcalix [4]arene-tetrakis(N,N-dimethylthioacetamide)) possesses a calix [4] arene scaffold bearing four electron-rich thioacetamide groups, forming a selective coordination cavity through Pb–S and Pb–N interactions [18,19]. This structure has been shown through molecular simulations to form highly stable complexes with Pb^2+^ [20], and is widely used in Pb(II)-selective sensors due to its high selectivity and sensitivity [20,21,22]. Moreover, the thioether groups of the ionophore can bind strongly to the AuNPs surface via Au–S interactions, allowing stable immobilization of the recognition molecules without compromising their activity.

Although various Fe_3_O_4_-Au-based sensors have been reported for heavy-metal detection, most of them employ simple core–shell or hybrid structures, where Au nanoparticles primarily act as conductive supports or immobilization platforms. These designs often exhibit limited exposure of active Au sites and insufficient interaction between recognition molecules and target ions. In contrast, our raspberry-like Fe_3_O_4_@PEI@AuNPs architecture offers several distinctive advantages: (i) the hierarchical “raspberry-like” structure provides a highly accessible and uniformly distributed Au surface for enhanced electron transfer and Pb^2+^ binding, (ii) the PEI layer ensures stable anchoring of AuNPs and Lead Ionophore IV, preventing leaching and improving interface robustness, and (iii) the combination of magnetic Fe_3_O_4_ and functionalized Au surface enhances pre-concentration efficiency under DPV conditions, resulting in higher sensitivity and selectivity compared to those of previously reported hybrid sensors. These aspects highlight the novelty of our work and distinguish it from existing research papers. In our “raspberry-like” Fe_3_O_4_@PEI@AuNPs nanostructure, Fe_3_O_4_ serves as the magnetic core, polyethylenimine (PEI) acts as a functional linker, and AuNPs are uniformly distributed on the surface. The sensor was fabricated on a screen-printed carbon electrode (SPCE) with an active working area of 2.64 mm^2^, offering a compact design, simple operation, and suitability for on-site measurements. Overall, this system demonstrates strong potential for real-world monitoring of Pb^2+^ contamination in polluted environments and the development of portable environmental sensing devices.

## 2. Materials and Methods

### 2.1. Chemicals and Reagents

Pb^2+^ standard solutions (1000 μg/mL), polyethyleneimine (PEI), Lead Ionophore IV (IONO), polyvinylpyrrolidone (PVP), sodium borohydride (NaBH_4_), Tetrahydrofuran (THF) were purchased from Sigma Aldrich, Burlington, MA, USA. Iron(III) Chloride Hexahydrate (FeCl_3_·6H_2_O), sodium acetate (NaAc), acid acetic (HAc), ethylene glycol (EG), tri-sodium citrate (HOC(COONa)(CH_2_COONa)_2_·2H_2_O) were obtained from Merck KGaA at Darmstadt, Germany; 2-[4-(2-Hydroxyethyl) piperazin-1-yl] ethane-1-sulfonic acid (HEPES) was the product of AKScientific, Union City, CA 94587, USA.

Deionized water (DW) and all other chemical reagents from different sources were of analytical grade.

### 2.2. Preparation of SPCE/Fe_3_O_4_@PEI@AuNPs_IONO-Modified Electrodes

#### 2.2.1. Synthesis of Fe_3_O_4_@PEI Nanoparticles

Fe_3_O_4_ nanoparticles were synthesized via a hydrothermal process in the presence of polyethylenimine (PEI). Initially, 0.68 g of FeCl_3_·6H_2_O, 1.0 g of polyethyleneimine (PEI), and 0.6 g of sodium acetate (NaAc) were accurately weighed. FeCl_3_·6H_2_O and PEI were each dissolved in a 10.0 mL ethylene glycol (EG) solution under magnetic stirring until clear. Sodium acetate (NaAc) was then added to the PEI/EG mixture, followed by thorough stirring to ensure complete dissolution. Finally, the FeCl_3_/EG solution was slowly poured into the PEI/NaAc/EG solution under continuous stirring to obtain a homogeneous reaction mixture.

The obtained solution was magnetically stirred at 60 °C for 20 min, during that the color gradually changed to dark brown, indicating the formation of iron oxide precursors. The resulting homogeneous solution was then transferred into a Teflon-lined stainless-steel autoclave and subjected to a hydrothermal reaction at 220 °C for 2 h. After cooling to room temperature, the product was collected and washed sequentially with ethanol and deionized water (DW) to remove unreacted species and residual impurities. The purified Fe_3_O_4_ nanoparticles were then dried under vacuum conditions for further use.

Subsequently, Fe_3_O_4_@PEI nanoparticles were prepared by surface modification of the synthesized Fe_3_O_4_ nanoparticles with PEI. 25.0 mg of Fe_3_O_4_ nanoparticles were ultrasonically dispersed in 40.0 mL of deionized water (DW) to obtain a uniform suspension. Separately, 1.5 g of polyethylenimine (PEI) was dissolved in 40.0 mL of DW by heating at 65 °C under magnetic stirring until complete dissolution. The Fe_3_O_4_ dispersion was then gradually added into the PEI solution (after removing the magnetic stir bar) under continuous agitation. The resulting mixture was subsequently diluted with 220.0 mL of DW and mechanically stirred at 65 °C and 170 rpm for 2 h to allow PEI to coat the nanoparticle’s surface. After the reaction, the obtained suspension was centrifuged and repeatedly washed with DW to remove excess PEI and unbound species. The final product was collected and designated as Fe_3_O_4_@PEI nanoparticles.

#### 2.2.2. Synthesis of Au@PVP Nanoparticles

Au@PVP nanoparticles were synthesized as follows: 3.5 mL of 30 mM HAuCl_4_ was added to 150.0 mL of deionized water under magnetic stirring for 1 min, followed by the addition of 5 mL of 38.8 mM sodium citrate and stirring for another 1 min. Subsequently, 1.0 mL of 0.75% NaBH_4_ (prepared in 38.8 mM sodium citrate) was introduced, and the mixture was stirred for 5 min. The flask was then covered with aluminum foil and kept undisturbed for 24 h. Next, 40.0 mL of 1.5% PVP solution was added, and the mixture was stirred for an additional 24 h. The product was collected by centrifugation at 12,000 rpm for 30 min, washed with DW to obtain Au@PVP nanoparticles.

#### 2.2.3. Attachment of Gold Nanoparticles (AuNPs)

10 mM HEPES buffer solution containing 1 mM polyvinylpyrrolidone (PVP) was prepared and adjusted to pH 7. Subsequently, Au@PVP nanoparticle dispersion was added to the buffer at a volume ratio of 1:20 (*v*/*v*). The Fe_3_O_4_@PEI nanoparticles (prepared as described in Section 2.1) were then gradually dispersed into the above solution at a ratio of 1/3 (mg/mL). The resulting mixture was sonicated for 30 min to enhance the attachment efficiency between the nanoparticles. Finally, the product was washed thoroughly with deionized water and dried under vacuum conditions. The obtained material was denoted as Fe_3_O_4_@PEI@AuNPs.

#### 2.2.4. Functionalization with Lead Ionophore IV

Magnetic nanoparticles (0.5 mg), previously coated with AuNPs in step 2.3, were dispersed in 1.0 mL of deionized (DI) water and sonicated with gentle pipette mixing in 10 min for uniform distribution. Subsequently, 300 μL of IONO solution (0.47% *w*/*v* in THF) was added dropwise to the nanoparticle dispersion under continuous sonication and pipette mixing for an additional 5 min to facilitate IONO immobilization on the particle surface. The resulting suspension was thoroughly washed with DI water to remove unbound IONO and then re-dispersed in 500 μL of DI water for downstream applications. Until further use, the nanoparticle suspension was sealed with parafilm and stored at 4 °C in the dark.

After fabricating and modifying the magnetic nanoparticles, we attached the particles to the electrode surface using the drop-coating method to create the SPCE/Fe_3_O_4_@PEI@AuNPs_IONO sensor.

### 2.3. Electrochemical Measurement

In the differential pulse voltammetry (DPV) measurements using the SPCE/Fe_3_O_4_@PEI@AuNPs_IONO sensor, the preconcentration (accumulation) of Pb^2+^ ions on the electrode surface plays a crucial role in enhancing the analytical signal. During this stage, Pb^2+^ ions in the solution are selectively captured and complexed by Lead Ionophore IV, resulting in their local enrichment. This accumulation process increases the local concentration of Pb^2+^ ions at the electrode interface, thereby amplifying the peak current intensity in the DPV measurements. Optimization of the accumulation potential and time is essential to achieve the desired sensitivity and detection limit, which reflects the performance of the three-dimensional “raspberry-like” Fe_3_O_4_@PEI@AuNPs nanostructure.

The accumulation of Pb^2+^ ions in the test samples was conducted using the chronoamperometry (CA) technique. The test solutions were diluted in acetate buffer with pH values of 4.8 and 5.7. 35 μL aliquot of the solution was placed to cover the three electrodes of the sensor, and a fixed potential was applied for a predetermined period. After the accumulation step, the electrode was rinsed with deionized water (DW) and subjected to DPV measurement. The acetate buffer was used as the supporting electrolyte, and its effect was examined at pH 4.8 and pH 5.7. The potential was scanned from −1.2 V to +0.5 V vs. Ag/AgCl with a step potential of 5 mV, a pulse amplitude of 50 mV, and a pulse duration of 50 ms. The peak current intensity in the DPV spectra increased linearly with Pb^2+^ concentration, allowing quantitative determination of Pb^2+^ ions based on the calibration curve of current (I_p_) versus concentration.

In this study, all experimental data were collected from three independent repeated measurements (*n* = 3) to ensure statistical reliability and reproducibility of the results. The linear regression equation was used to calculate the limit of detection (LOD) according to the formula LOD = 3σ/slope, where σ is the standard deviation of the intercept, and slope is the gradient of the calibration curve.

## 3. Results and Discussion

### 3.1. Structural Characterization of Raspberry-like Fe_3_O_4_@PEI@AuNPs and Ionophore Modification

#### 3.1.1. TEM Images

**Figure 1:**
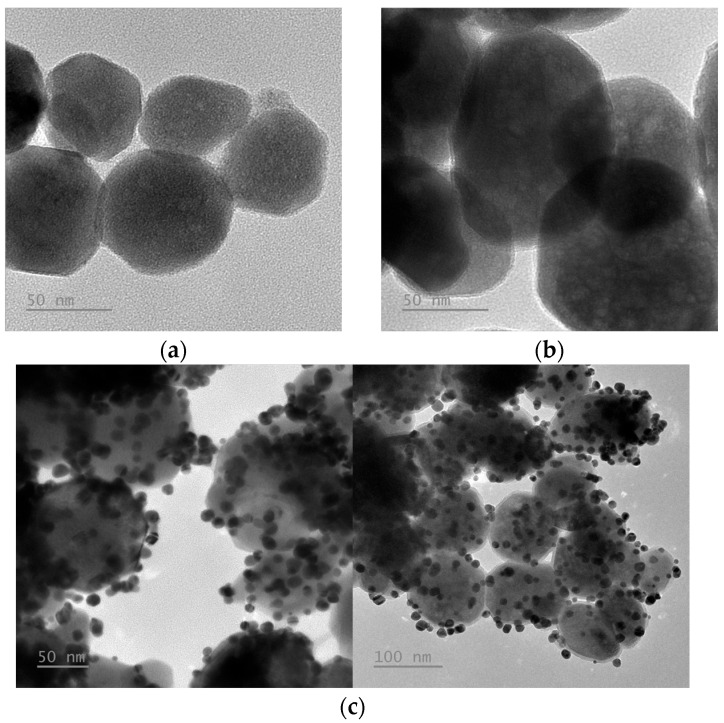
TEM images of (**a**) Fe_3_O_4_; (**b**) Fe_3_O_4_@PEI; and (**c**) Fe_3_O_4_@PEI@AuNPs, showing a raspberry-like core–shell structure, where gold nanoparticles are densely attached to the PEI-modified magnetic Fe_3_O_4_ core.

Figure 1 presents the TEM images of Fe_3_O_4_, Fe_3_O_4_@PEI and Fe_3_O_4_@PEI@AuNPs nanoparticles. The Fe_3_O_4_ particles exhibit a nearly spherical morphology with a uniform size distribution of approximately 60–80 nm, featuring smooth surfaces and clear separation between individual particles. After coating with PEI and anchoring AuNPs, the particle surfaces become rougher, displaying numerous dark spots with diameters of 10–15 nm densely distributed around the magnetic cores. The appearance of these high-contrast spots confirms the successful attachment of gold nanoparticles onto the Fe_3_O_4_ surface via the PEI interlayer, resulting in the formation of a distinctive “raspberry-like” nanostructure consistent with the intended material design.

#### 3.1.2. Raman Spectroscopy

**Figure 2:**
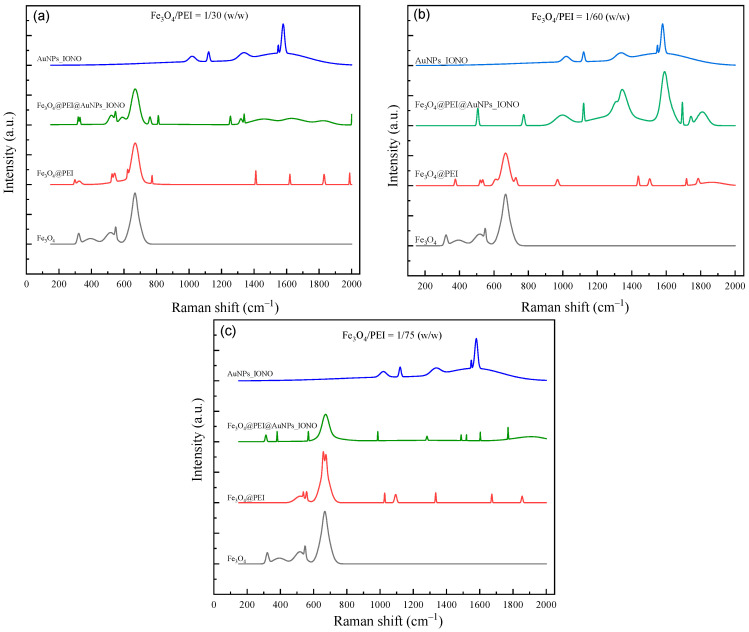
Raman spectra of Fe_3_O_4_@PEI samples synthesized at different Fe_3_O_4_:PEI weight ratios, including (**a**) 1:30, (**b**) 1:60, and (**c**) 1:75.

The Raman spectra of the magnetic nanoparticles at various surface modification stages for three different Fe_3_O_4_/PEI mass ratios (1/30, 1/60, and 1/75) are presented in Figure 2. For the pristine Fe_3_O_4_ sample, a strong characteristic band appears at approximately 670 cm^−1^, corresponding to the symmetric stretching vibration of Fe–O bonds within the spinel lattice structure of Fe_3_O_4_, confirming the typical magnetite phase. After coating with PEI, the intensity of this band slightly decreases, accompanied by the appearance of weak vibrational bands in the range of 1350–1600 cm^−1^, which are attributed to the stretching and bending vibrations of amine (-NH_2_) groups and C-N bonds, characteristic of PEI. This observation confirms the successful formation of an organic polymer layer on the surface of the magnetic nanoparticles.

Upon the subsequent functionalization with gold nanoparticles (AuNPs) and ionophore molecules (IONO), the Raman spectra of Fe_3_O_4_@PEI@AuNPs_IONO exhibit additional distinct peaks at around 1120, 1344, and 1590 cm^−1^, corresponding to the C=S, C=C, and C-N vibrational modes in the structure of Lead Ionophore IV. These features are characteristic of the aromatic ring and thiocarbamoyl functional groups in the ionophore, indicating that the immobilization of the ionophore onto the material surface was successful. Notably, compared to the spectrum of pure AuNPs_IONO, the characteristic peaks of the ionophore in Fe_3_O_4_@PEI@AuNPs_IONO show significantly enhanced intensity and a slight blue shift, implying the formation of strong chemical interactions between the thiol (-SH) groups of the ionophore and the gold surface. This confirms the creation of a well-ordered and stable IONO–SH monolayer on the AuNPs surface.

When comparing the three Fe_3_O_4_/PEI ratios, the sample with a ratio of 1/60 displays the most well-defined and balanced Raman features, suggesting a uniform and effective PEI coating that provides a favorable surface for homogeneous AuNP immobilization. This facilitates the formation of an ordered and densely packed IONO–SH monolayer. In contrast, the 1/30 ratio sample shows a pronounced Fe–O band (~670 cm^−1^) but a strong spectral background, while the characteristic ionophore bands (C=S, C-N, C=C) appear weak or obscured. This behavior can be attributed to the excessive PEI content, which leads to the formation of a thick polymer layer that masks the Fe_3_O_4_ surface and hinders AuNPs deposition as well as ionophore binding. Conversely, at the 1/75 ratio, the Fe-O band intensity increases, but the organic-related peaks diminish significantly, indicating that the PEI coverage is insufficient to fully encapsulate the magnetic cores. As a result, AuNPs are unevenly distributed and ionophore immobilization becomes less effective.

Overall, the Raman analysis reveals that the Fe_3_O_4_/PEI ratio of 1/60 is optimal, ensuring a stable polymer coating, high AuNPs density, and the successful formation of a robust IONO–SH monolayer. These structural characteristics provide ideal conditions for the selective recognition of Pb^2+^ ions by the hybrid nanomaterial system.

### 3.2. Optimization of Electrochemical Conditions for Pb^2+^ Detection

To achieve effective electrochemical detection of Pb^2+^ ions, the supporting electrolyte and pH conditions were first optimized. Acetate buffer was selected because its weakly acidic environment helps maintain Pb^2+^ in the free ionic state, preventing the formation of insoluble Pb(OH)_2_ or other hydrolyzed species that often occur under neutral or basic conditions. Moreover, acetate ions interact only weakly with Pb^2+^, thus avoiding competitive complexation with the ionophore. The slightly acidic medium also enhances charge-transfer kinetics and ensures that the thiol-based ionophore layer remains stable during measurement. Therefore, acetate buffers with pH values of 4.8 and 5.7 were chosen for comparison.

**Figure 3:**
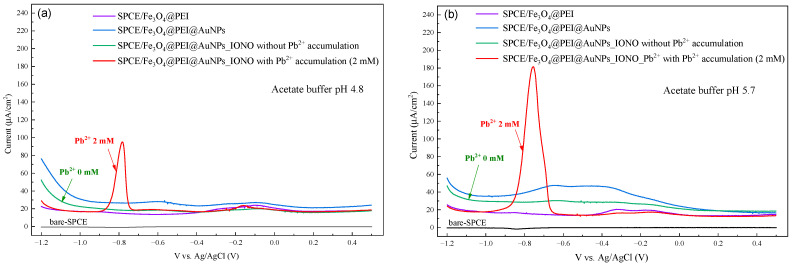
DPV for Pb^2+^ detection recorded in acetate buffer (**a**) pH 4.8 and (**b**) pH 5.7 at different stages of electrode preparation.

Differential pulse voltammetry (DPV) measurements were performed after Pb^2+^ accumulation on a series of electrodes: bare SPCE, SPCE/Fe_3_O_4_@PEI@AuNPs, and SPCE/Fe_3_O_4_@PEI@AuNPs_IONO. As shown in Figure 3, only the SPCE/Fe_3_O_4_@PEI@AuNPs_IONO electrode exhibited a distinct reduction peak at approximately −0.78 V vs. Ag/AgCl, while no clear peaks were observed for the other electrodes. This indicates that the ionophore layer plays a key role in selectively capturing Pb^2+^ ions and facilitating their electrochemical reduction. Furthermore, the DPV peak current recorded in acetate buffer at pH 5.7 was significantly higher than that at pH 4.8, suggesting that a moderately acidic environment favors the ionophore—Pb^2+^ interaction and promotes more efficient electron transfer at the electrode surface. Consequently, pH 5.7 was selected as the optimal electrolyte condition for subsequent experiments. The choice of this pH value is fully consistent with previous studies [23,24,25,26]. These results confirm that the raspberry-like Fe_3_O_4_@PEI@AuNPs nanostructure functionalized with Lead Ionophore IV retains high sensitivity and selectivity toward Pb^2+^ even under mildly acidic conditions, demonstrating the robustness and stability of the sensor interface developed in this work.

**Figure 4:**
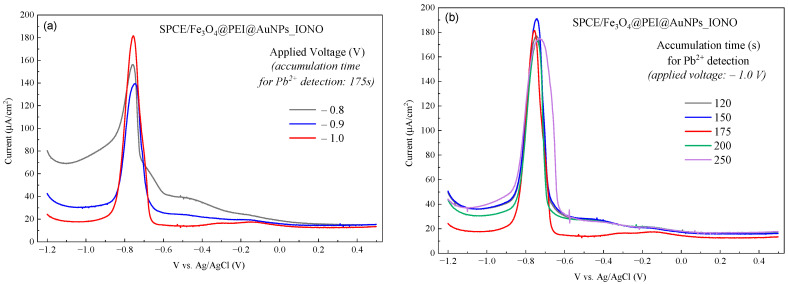
DPV responses of the SPCE/Fe_3_O_4_@PEI@AuNPs_IONO sensor recorded under different Pb^2+^ accumulation conditions: (**a**) effect of accumulation potential (−0.8, −0.9, and −1.0 V) at a fixed accumulation time of 175 s, and (**b**) effect of accumulation time (120–250 s) at a fixed potential of −1.0 V. The Pb^2+^ concentration was 2.0 mM. The peak current increased with both accumulation potential and time, reaching the highest response at −1.0 V for 175 s, indicating that these parameters provide the optimal conditions for Pb^2+^ preconcentration on the electrode surface.

The accumulation potential and time were further optimized to maximize the DPV response. As shown in Figure 4a, the reduction peaks current of Pb^2+^ strongly depends on the applied accumulation potential. When the accumulation potential was varied from −0.8 V to −1.0 V, the peak current gradually increased and reached its maximum at −1.0 V, indicating that this potential provides the most efficient reduction and adsorption of Pb^2+^ onto the modified electrode surface. At less negative potentials, the driving force for Pb^2+^ reduction is insufficient, leading to limited ion adsorption and a weaker current response.

Similarly, the influence of accumulation time on the DPV signal was examined (Figure 4b). As the accumulation time increased from 120 s to 175 s, the peak current rose correspondingly, reflecting the progressive buildup of Pb^2+^ ions on the electrode surface. However, when the accumulation time exceeded 200 s, the current intensity tended to level off and slightly decrease, likely due to surface saturation or partial re-dissolution of Pb^2+^ species. Based on these observations, the optimal accumulation conditions for Pb^2+^ detection on the SPCE/Fe_3_O_4_@PEI@AuNPs_IONO electrode were determined to be an accumulation potential of −1.0 V and an accumulation time of 175 s, ensuring both high sensitivity and stable DPV responses.

### 3.3. Analytical Performance and Selectivity of the Developed Sensor

**Figure 5:**
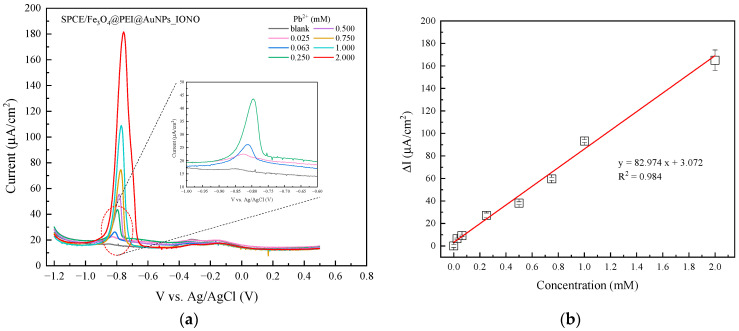
(**a**) DPV responses of the SPCE/Fe_3_O_4_@PEI@AuNPs_IONO sensor toward various concentrations of Pb^2+^; and (**b**) the corresponding calibration plot of peak current (I_p_) versus Pb^2+^ concentration. The accumulation of Pb^2+^ ions was performed at −1.0 V (vs. Ag/AgCl) for 175 s prior to each DPV measurement.

The DPV response of the SPCE/Fe_3_O_4_@PEI@AuNPs_IONO sensor (Figure 5) exhibits a distinct reduction peak at approximately −0.78 V vs. Ag/AgCl, corresponding to the electrochemical reduction of Pb^2+^ to metallic Pb^0^ on the electrode surface. As the Pb^2+^ concentration in the solution increases from 0.025 mM to 2.000 mM, the peak current intensity rises linearly, indicating a clear and stable sensor response to varying lead ion concentrations (see Table 1). The enhancement of the current signal can be attributed to the synergistic effects of the electrode coating components: Fe_3_O_4_ provides magnetic conductivity and a large surface area; PEI, containing positively charged amine groups, facilitates Pb^2+^ adsorption through electrostatic interactions; while AuNPs enhance electron transfer kinetics, thereby improving the overall sensitivity of the sensing system.

From the DPV results at different Pb^2+^ concentrations, the average peak current values obtained from three independent sensors at each concentration point were used to construct the calibration curve (∆I—[Pb^2+^]). The calibration plot demonstrates a good linear correlation between the current intensity and Pb^2+^ concentration in the range of 0.025–2.000 mM, described by the equation:

y = 82.974x + 3.072, R^2^ = 0.984. (1)

where y represents the peak current density (μA/cm^2^) and x is the Pb^2+^ concentration (mM). The high correlation coefficient (R^2^ = 0.984) confirms excellent measurement reproducibility and reliable quantification performance of the electrode. Based on the calibration equation, the sensitivity of the sensor toward Pb^2+^ ions were determined to be 82.974 μA.mM^−1^·cm^−2^.

The limit of detection (LOD) and limit of quantification (LOQ) were calculated according to the standard equations:

(2) $$LOD=\;\frac{3.3\sigma_{blank}}{m},\;LOQ=\;\frac{10\sigma_{blank}}{m}$$

where $\sigma_{blank}$ is the standard deviation of the blank signal and m is the slope of the calibration curve.

Based on the DPV measurements of the blank sample, the average background current was 16.5 μA, with a standard deviation $\sigma_{blank}$ = 1.41 μA. Using the calibration equation above, the LOD and LOQ values of the sensor were calculated to be 0.056 mM and 0.170 mM Pb^2+^, respectively. The repeatability of the Fe_3_O_4_@PEI@AuNPs-based sensor was assessed by intra-electrode and inter-electrode measurements at 1 mM Pb^2+^. The %RSD for three consecutive measurements using the same electrode was 1.16%, while the %RSD for three independently prepared electrodes was 4.11%, indicating satisfactory reproducibility and stability of the sensor.

### 3.4. Selectivity of the Developed Pb^2+^ Sensor

**Figure 6:**
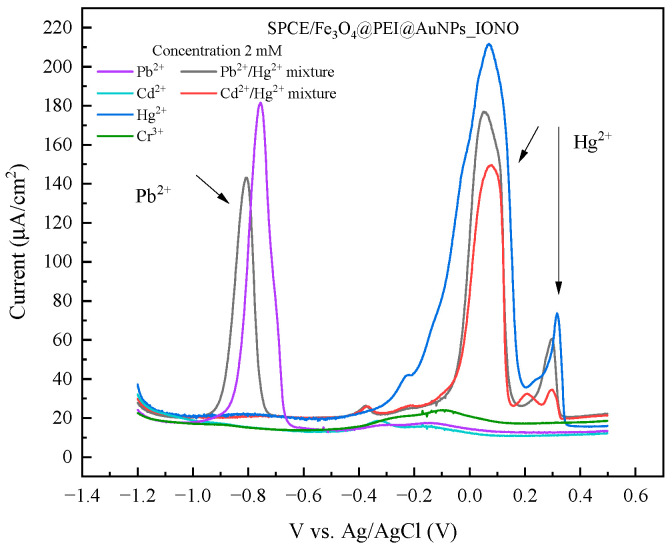
DPV responses of the SPCE/Fe_3_O_4_@PEI@AuNPs_IONO sensor toward different metal ions (Pb^2+^, Cr^3+^, Cd^2+^, and Hg^2+^) at a concentration of 1.0 mM under the optimized accumulation conditions (−1.0 V for 175 s). The sensor exhibited the strongest response toward Pb^2+^, while a noticeable signal was also observed for Hg^2+^, which can be attributed to the partial exposure of the SPCE surface and the incomplete coverage of AuNPs by the IONO monolayer. This result indicates a predominant selectivity for Pb^2+^, with some cross-sensitivity to Hg^2+^.

The selectivity of the SPCE/Fe_3_O_4_@PEI@AuNPs_IONO sensor was evaluated by measuring its DPV responses in the presence of potentially interfering metal ions, including Cd^2+^, Hg^2+^, and Cr^3+^, as well as binary mixtures containing Pb^2+^ with these ions. Figure 6 presents the DPV responses of the SPCE/Fe_3_O_4_@PEI@AuNPs_IONO sensor toward different metal ions at a concentration of 1.0 mM under the optimized accumulation conditions (−1.0 V for 175 s). The results show that for other cations such as Cd^2+^ and Cr^3+^, no discernible peaks were observed in the DPV profiles, indicating no significant interference within the potential region where Pb^2+^ is detected. This confirms the strong selectivity of the ionophore toward Pb^2+^ ions.

In contrast, for Hg^2+^ two distinct peaks appeared at approximately +0.09 V and +0.32 V, with the lower-potential peak showing an intensity about three to four times higher than the second one. When analyzing binary mixtures of Pb^2+^/Hg^2+^, three peaks were observed at −0.80 V (Pb^2+^) and around +0.05 V and +0.30 V (corresponding to Hg^2+^), whereas the Cd^2+^/Hg^2+^ mixture showed only two peaks near +0.07 V and +0.30 V. This indicates that the Hg^2+^ signals persist regardless of the presence of Pb^2+^ or Cd^2+^. These observations suggest that the two peaks associated with Hg^2+^ are not due to specific interactions between Hg^2+^ and the ionophore but rather originate from unmodified or partially exposed surface sites—namely, the bare SPCE carbon regions or uncovered AuNPs on the raspberry-like structure—where Hg^2+^ can be adsorbed and undergo redox (stripping) processes at distinct potentials.

This hypothesis was further supported by control experiments using an SPCE electrode pre-deposited with a uniform layer of Au nanoparticles via cyclic voltammetric electrodeposition prior to ionophore immobilization. After forming a more homogeneous Au surface and subsequently attaching the ionophore SAM, the DPV spectra exhibited only one Hg^2+^ peak at approximately +0.05 to +0.09 V, while the higher-potential peak (~+0.30 V) was greatly reduced or disappeared. Meanwhile, the Pb^2+^ peak remained stable at −0.77 to −0.79 V, and Cd^2+^ continued to show no detectable signal. The disappearance of the ~+0.30 V peak after the formation of a uniform Au layer and complete ionophore coverage suggests that this peak is most likely related to heterogeneous surface regions (exposed AuNPs or inert carbon areas) on the SPCE/Fe_3_O_4_@PEI@AuNPs_IONO electrode. These sites could induce two types of electrochemical interactions with Hg^2+^, such as underpotential deposition (UPD) forming Au–Hg amalgams, or adsorption/oxidation on carbon domains, resulting in distinct stripping potentials and current intensities. The higher intensity of the lower-potential peak (+0.05 to +0.09 V) indicates that this process is kinetically more favorable and occurs at more abundant sites, consistent with its persistence after SAM coverage.

Importantly, the unchanged Pb^2+^ peak potential (~−0.78 V) in the presence of Hg^2+^ demonstrates that the ionophore maintains its strong selectivity and binding affinity for Pb^2+^, while the residual Hg^2+^ peaks confirm that preconcentration and reduction of Hg^2+^ occur independently at the remaining uncoated sites. Possible physico-chemical explanations for the two Hg^2+^ peaks include: (1) different surface domains (Au vs. carbon, or AuNPs of varying size/aggregation states) resulting in distinct UPD and bulk stripping potentials; (2) coexistence of two chemical states of Hg (e.g., adsorbed vs. weakly bound species); and (3) variation in AuNPs conductivity and local potential distribution that shifts the oxidation potential of Hg^2+^ species.

**Figure 7:**
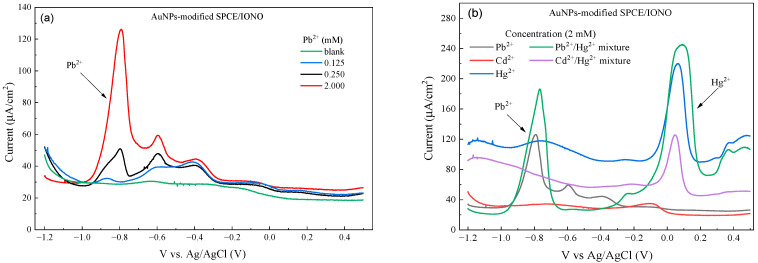
(**a**) DPV spectra of the AuNPs-modified SPCE/IONO electrode in the presence of different Pb^2+^ concentrations, showing the characteristic oxidation peak of Pb^2+^ with moderate signal broadening at higher concentrations; and (**b**) DPV responses of the same electrode toward individual Pb^2+^, Cd^2+^, and Hg^2+^ ions, and binary mixtures of Pb^2+^/Hg^2+^ and Cd^2+^/Hg^2+^. The sensor exhibits specific affinity for Pb^2+^ ions as designed, while an additional response to Hg^2+^ is also observed, attributed to the partial exposure of the SPCE surface and incomplete IONO coverage over the AuNP layer.

To further investigate the origin of the Hg^2+^ peaks observed in the previous selectivity study, DPV measurements were performed on an AuNPs-modified SPCE electrode (AuNPs-modified SPCE/IONO) with a more uniform gold layer prior to ionophore immobilization. As shown in Figure 7a, the characteristic Pb^2+^ peak remained clearly visible at approximately −0.78 V (vs. Ag/AgCl), and its peak current increased proportionally with Pb^2+^ concentration from 0.125 to 2.0 mM, demonstrating a clear dose-dependent response.

When assessing selectivity toward Hg^2+^ and other heavy metal ions (Figure 7b), only a single weak Hg^2+^ peak appeared at around +0.05 to +0.09 V, whereas the higher-potential peak (~+0.30 V) observed on the SPCE/Fe_3_O_4_@PEI@AuNPs_IONO electrode was absent. This confirms that the additional Hg^2+^ peak seen previously originated from uncoated or exposed sites on the original raspberry-like AuNPs or the bare SPCE surface, which allowed Hg^2+^ to adsorb and undergo redox reactions independently of the ionophore. The Pb^2+^ peak position remained unchanged in the presence of Hg^2+^, further confirming that the ionophore preserves high selectivity toward Pb^2+^ even in the presence of other competing ions.

These results demonstrate that a uniform AuNPs layer combined with a complete ionophore SAM not only improves the reproducibility of the DPV signal but also minimizes interference from Hg^2+^ by eliminating heterogeneous “exposed” sites, thereby highlighting the critical role of AuNPs in achieving selective Pb^2+^ detection.

**Figure 8:**
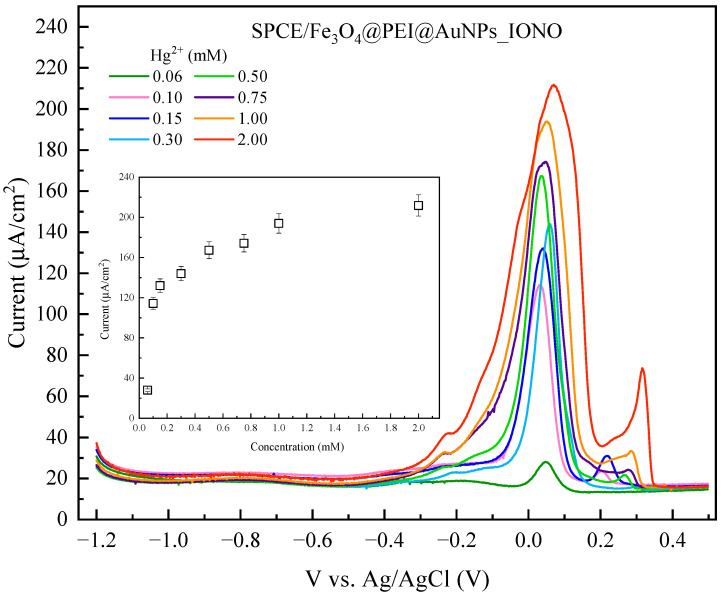
DPV responses of the SPCE/Fe_3_O_4_@PEI@AuNPs_IONO electrode with the addition of different concentrations of Hg^2+^ ions, and the corresponding calibration plot of the peak current versus Hg^2+^ concentration. The accumulation of Hg^2+^ ions was performed at a potential of −1.0 V (vs. Ag/AgCl) for 175 s prior to each DPV measurement. Although the IONO ligand was designed to selectively recognize Pb^2+^ ions, a measurable response toward Hg^2+^ was still observed, likely due to the partially exposed SPCE surface and the incomplete coverage of AuNPs–IONO on the electrode.

After evaluating the selectivity toward Pb^2+^, the electrochemical response of the SPCE/Fe_3_O_4_@PEI@AuNPs_IONO sensor toward Hg^2+^ was further investigated, and a calibration curve was constructed. As shown in Figure 8, the peak current at around +0.09 V increased progressively with Hg^2+^ concentration, indicating that the sensor is capable of detecting Hg^2+^. A second peak at approximately +0.32 V only appeared when the Hg^2+^ concentration exceeded 0.15 mM. Notably, the DPV peaks for Hg^2+^ exhibited broader and less defined shapes compared to the sharp, symmetrical peak observed for Pb^2+^. The asymmetry and broadening of the Hg^2+^ peaks can be attributed to multiple factors: (1) heterogeneous adsorption and electron-transfer kinetics on partially exposed AuNPs or SPCE surface sites; (2) coexistence of different Hg^2+^ species (e.g., adsorbed vs. weakly coordinated), which oxidize at slightly different potentials; and (3) slower charge-transfer dynamics for Hg^2+^ at the ionophore-functionalized surface. These characteristics result in a peak with a “foot” or shoulder, and a less sharp apex, in contrast to the well-defined Pb^2+^ response.

Overall, despite the broadened and asymmetric peak shapes, the SPCE/Fe_3_O_4_@PEI@AuNPs_IONO sensor demonstrates a clear concentration-dependent response toward Hg^2+^, allowing quantitative analysis, though with lower resolution and selectivity compared to Pb^2+^.

In conclusion, the SPCE/Fe_3_O_4_@PEI@AuNPs_IONO sensor is capable of detecting both Pb^2+^ and Hg^2+^ simultaneously. The key to achieving reliable dual-ion detection lies in the controlled fabrication of the raspberry-like Fe_3_O_4_@PEI@AuNPs structures, with well-defined regions functionalized with the ionophore (IONO) for Pb^2+^ recognition and deliberately unmodified AuNPs for Hg^2+^ preconcentration. By optimizing the reproducibility and distribution of these functionalized and non-functionalized sites, the sensor can provide selective and quantitative responses for both metal ions, while minimizing peak overlap and maintaining signal stability. This approach highlights the potential of using spatially controlled nanoarchitectures to develop versatile multi-ion electrochemical sensors.

**Figure 9:**
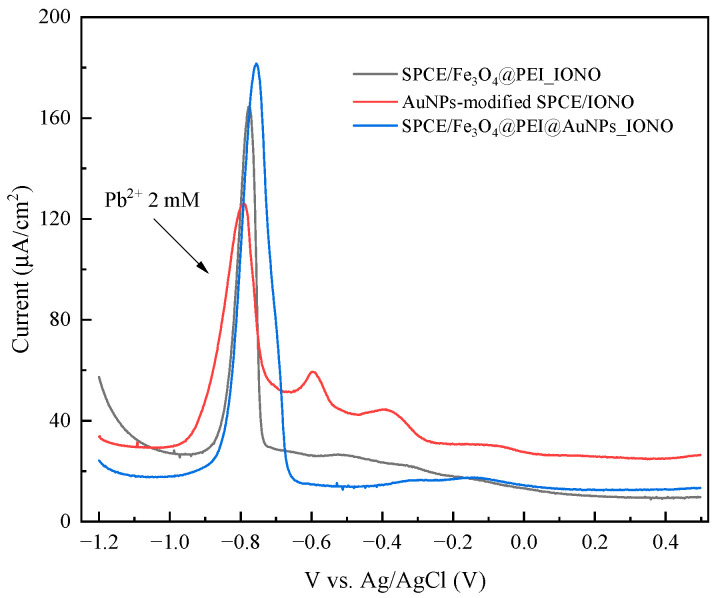
DPV responses of AuNPs-modified SPCE/IONO, SPCE/Fe_3_O_4_@PEI/IONO, and SPCE/Fe_3_O_4_@PEI@AuNPs/IONO in acetate buffer pH 5.7 containing 2.0 mM Pb^2+^.

To further elucidate the role of each material component in enhancing Pb^2+^ sensing performance, comparative DPV measurements were carried out using three electrode configurations: AuNPs-modified SPCE/IONO; SPCE/Fe_3_O_4_@PEI_IONO; SPCE/Fe_3_O_4_@PEI@AuNPs_IONO (see Figure 9). The corresponding peak currents recorded at −0.81 V were 95, 165, and 181 μA/cm^2^, respectively. The substantial increase observed for SPCE/Fe_3_O_4_@PEI_IONO (approximately 1.7-fold higher than AuNPs-modified SPCE/IONO) indicates that the Fe_3_O_4_ core and PEI coating play dominant roles in the preconcentration of Pb^2+^. The amine-rich PEI layer introduces abundant -NH_2_ and -NH groups, which become partially protonated under mildly acidic conditions (pH 5.7), leading to strong electrostatic attraction and coordination with Pb^2+^ species. Simultaneously, the Fe_3_O_4_ nanoparticles contribute to analyte enrichment by increasing the specific surface area and generating localized magnetic effects that promote ionic diffusion and accumulation near the electrode interface. This synergistic interaction between Fe_3_O_4_ and PEI markedly enhances Pb^2+^ capture prior to electrochemical reduction. When AuNPs were subsequently decorated around the Fe_3_O_4_@PEI matrix to form a raspberry-like Fe_3_O_4_@PEI@AuNPs structure, the current response further increased to 181 μA/cm^2^ (about 1.1 times higher than Fe_3_O_4_@PEI_IONO). This improvement demonstrates that AuNPs significantly accelerate interfacial charge transfer between the ionophore-Pb^2+^ complex and the electrode surface, owing to their excellent electrical conductivity and strong Au–S bonding interactions with Lead Ionophore IV. Moreover, the uniform distribution of AuNPs across the PEI-modified Fe_3_O_4_ surface creates a highly accessible network of electroactive sites, minimizing charge transfer resistance and stabilizing the signal response during repeated scans.

Overall, these results confirm that the Pb^2+^ sensing mechanism arises from a dual cooperative effect: (i) PEI and Fe_3_O_4_ jointly enable efficient preconcentration of Pb^2+^ ions at the sensing interface through electrostatic attraction and magnetic enrichment, and (ii) AuNPs facilitate rapid electron transfer and maintain the integrity of the ionophore layer. The resulting Fe_3_O_4_@PEI@AuNPs_IONO hybrid thus exhibits higher sensitivity, reproducibility, and operational stability compared with the single-component systems.

As summarized in Table 2, the SPCE/Fe_3_O_4_@PEI@AuNPs_IONO electrode developed in this work exhibits a wide linear detection range for Pb^2+^ (0.025–2.00 mM), which surpasses those of most previously reported sensors. Although its detection limit (0.056 mM) is relatively higher, the electrode demonstrates excellent adaptability for samples containing varying Pb^2+^ concentrations. This broad range, combined with the simplicity, low cost, and disposability of the SPCE-based platform, highlights the potential of the proposed core–shell multilayer nanostructure for practical environmental monitoring applications.

Based on the electrochemical characterization, the SPCE/Fe_3_O_4_@PEI@AuNPs_IONO sensor demonstrates excellent performance for Pb^2+^ detection, exhibiting sharp, well-defined DPV peaks and a clear concentration-dependent response. In contrast, the DPV response for Hg^2+^ is broad, asymmetric, and only shows a secondary peak at relatively high concentrations, reflecting slower electron-transfer kinetics and heterogeneous adsorption on exposed AuNPs or SPCE sites. These characteristics make Hg^2+^ detection less reproducible and more difficult to quantify accurately. Focusing the sensor on Pb^2+^ detection allows for higher selectivity, more reliable calibration, and robust signal reproducibility. Moreover, from an environmental monitoring perspective, Pb^2+^ is a major contaminant of concern in water systems, whereas Hg^2+^ is typically present at lower concentrations. Therefore, optimizing the sensor for selective Pb^2+^ detection ensures practical applicability, reliable analytical performance, and consistent results, while minimizing interference from other metal ions.

## 4. Conclusions

In this study, we developed a highly selective and sensitive electrochemical sensor for Pb^2+^ based on SPCE modified with raspberry-like Fe_3_O_4_@PEI@AuNPs functionalized with a thiol-based ionophore (Lead Ionophore IV). TEM and Raman analyses confirmed the successful fabrication of the nanostructures and the optimal composition for uniform AuNPs deposition and effective ionophore immobilization. The sensor exhibited a well-defined DPV peak at −0.78 V, with a clear concentration-dependent response to Pb^2+^, achieving a low limit of detection (LOD) of 0.056 mM. Optimization of electrochemical conditions, including accumulation potential and time, further enhanced sensitivity. Selectivity studies confirmed that the ionophore-functionalized electrode maintains excellent discrimination against other heavy metal ions, including Cd^2+^, Hg^2+^, and Cr^3+^, with any interference from Hg^2+^ attributed to exposed AuNP or SPCE sites rather than ionophore interactions. Although the sensor can respond to Hg^2+^ under specific conditions, its broad and asymmetric DPV signals make quantitative detection less reliable, suggesting that Pb^2+^ should remain the primary target. Overall, this sensor demonstrates great potential for practical applications in environmental monitoring, particularly for detecting Pb^2+^ contamination in water and other ecological systems, combining high selectivity, sensitivity, and reproducibility.

## Figures and Tables

**Table 1:** Peak current response (I_p_) of SPCE/Fe_3_O_4_@PEI@AuNPs_IONO electrodes toward different Pb^2+^ concentrations. The differential current (∆I = I_p_ − I_blank_) was used for calibration curve construction under accumulation potential of −1.0 V for 175 s.

Pb^2+^ Concentration (mM)	$I_{p}\;(\mu$A/cm^2^)	I_blank_	∆I = I_p_ − I_blank_
0	16.49137	16.49137	0
0.025	22.82193	16.49137	6.33057
0.063	25.56133	16.49137	9.06997
0.250	43.6186	16.49137	27.12723
0.500	54.5018	16.49137	38.01043
0.750	76.07503	16.49137	59.58367
1.000	109.756	16.49137	93.26463
2.000	181.596	16.49137	165.10463

**Table 2:** Comparison of the performance of SPCE/Fe_3_O_4_@PEI@AuNPs_IONO electrode with other electrodes tested for the determination of Pb^2+^ ions in the standard solutions.

Electrode	Heavy Metal Ions	LOD (ppm)	Linear Range (ppm)	Ref.
MWCNT/CS/Pb^2+^ ionophore IV/Au	Pb^2+^	8 × 10^−5^	0.001–0.1	[22]
G-COOH-MWCNTs/ZnO/GCE	Pb^2+^ Cd^2+^	5.35 × 10^−4^ 3.54 × 10^−4^	0.025–0.45	[27]
GC/NHAP/ionophore/Nafion	Pb^2+^	2.07 × 10^−4^	0.001–0.166	[28]
SPCE/PANI-PDTDA	Pb^2+^ Cd^2+^	0.0352 0.0601	2 × 10^−4^–207 2 × 10^−4^–207	[29]
Fe_3_O_4_@Citrate/GCE	Pb^2+^	0.0622	0.104–3.11	[25]
Fe_3_O_4_/GN/GE/GCE	Pb^2+^	2.55 × 10^−9^	2.07 × 10^−7^–1.04 × 10^−4^ 1.04 × 10^−4^–0.207	[30]
SPCE/Fe_3_O_4_@PEI@AuNPs_IONO	Pb^2+^	11.6 ppm (0.056 mM)	5.18–414.4 ppm (0.025–2.00 mM)	This work

## Data Availability

The original contributions presented in this study are included in the article. Further inquiries can be directed to the corresponding authors.

## Abbreviations

The following abbreviations are used in this manuscript:

HEPES	2-[4-(2-Hydroxyethyl) piperazin-1-yl] ethane-1-sulfonic acid
HAc	Acid Acetic
CA	Chronoamperometry
DW	Deionized Water
DPV	Differential pulse voltammetry
EG	Ethylene Glycol
IONO	Lead Ionophore IV
PEI	Polyethyleneimine
PVP	Polyvinylpyrrolidone
NaAc	Sodium Acetate
THF	Tetrahydrofuran
